# Chordin-like 1, a bone morphogenetic protein-4 antagonist, is upregulated by hypoxia in human retinal pericytes and plays a role in regulating angiogenesis

**Published:** 2008-06-20

**Authors:** Rosemary Kane, Catherine Godson, Colm O’Brien

**Affiliations:** 1 School of Medicine and Medical Science, UCD Conway Institute and UCD Diabetes Research Centre, University College Dublin, Belfield, Dublin 4, Ireland; 2 Institute of Ophthalmology, Mater Misericordiae Hospital, Eccles Street, Dublin 7, Ireland

## Abstract

**Purpose:**

Pericytes play a specialized role in regulating angiogenesis and vascular function by providing vascular stability and controlling endothelial cell proliferation. Disorders in pericyte function and pericyte-endothelial interaction have been observed in several disease states including tumor angiogenesis and diabetic microangiopathy. In ischemic retinal disease, hypoxia is a potent driver of retinal angiogenesis. This study investigated the effects of hypoxia on retinal pericyte gene expression, and demonstrates a role in angiogenesis regulation for the hypoxia driven gene, chordin-like 1 (*CHL-1*).

**Methods:**

In the current studies, we investigated hypoxia-induced gene expression in human retinal pericytes and found that expression of CHL-1, a member of the bone morphogenetic protein (BMP) superfamily, is upregulated by hypoxia. We investigated regulation of *CHL-1* expression and the ability of CHL-1 to antagonize the antiangiogenic properties of BMP-4 using a human cell-based angiogenesis assay.

**Results:**

We report that hypoxia induced hypoxia inducible factor-1α-driven expression of CHL-1. Both CHL-1 and BMP-4 were secreted from human retinal pericytes. We found that CHL-1 complexes with BMP-4 to antagonize the antiangiogenic effects of BMP-4, and that BMP-4 and vascular endothelial growth factor (VEGF) co-regulate angiogenesis.

**Conclusions:**

We propose that hypoxia-induced upregulation of CHL-1 alters the homeostatic balance between BMP-4 and VEGF to synergize with VEGF in driving retinal angiogenesis.

## Introduction

Diabetic retinopathy is a microvascular complication of both Type 1 and Type 2 diabetes and is characterized by increased tissue ischemia, angiogenesis, and permeability. This microvascular disease is a major cause of blindness in the working age population [1]. The development of diabetic retinopathy reflects the convergence of hemodynamic and metabolic insults, including hyperglycemia, in susceptible individuals.

Vascular endothelial growth factor (VEGF) is known to be induced by hypoxia and may mediate hypoxia-induced angiogenesis [2]. VEGF alone is sufficient to produce many of the vascular abnormalities common to diabetic retinopathy and other ischemic retinopathies, such as hemorrhage, edema, venous beading, capillary occlusion with ischemia, microaneurysm formation, and intraretinal vascular proliferation [3].

Retinal pericytes are smooth muscle-like cells with attenuated processes enveloping the abluminal surface of microvessels and sharing a common basement membrane with the underlying endothelium (reviewed in [4]). Pericytes express alpha-smooth muscle actin and have thus been implicated to have a contractile function [5]. They are proposed to regulate microvascular angiogenesis and synthesize components of the vascular basement membrane [6,7]. Pericytes have been demonstrated to be involved in the regulation of endothelial cell number and morphology and microvessel architecture [8].

In this study we present novel findings that chordin-like 1 (CHL-1), a bone morphogenetic protein (BMP) antagonist [9], is upregulated by hypoxia in human retinal pericytes, and that its expression is driven by hypoxia inducible factor-1α (HIF-1α). CHL-1 has previously been reported to be expressed in the developing retina [9], but has not yet been associated with diabetic or ischemic retinopathy. BMP-4 has been implicated in angiogenesis through a VEGF-dependent mechanism [10]. We therefore propose that CHL-1 expression by human retinal pericytes in response to hypoxia may play an important role in regulating retinal angiogenesis through modulation of BMP-4 actions on endothelial cells.

## Methods

### Cell culture and hypoxia

Primary cultures of human retinal pericytes (hRPC) were obtained from Cambrex (Nottingham, UK) and were cultured in MCDB131 medium (Invitrogen, Paisley, Scotland) supplemented with 2 mM L-glutamine 50 U/ml penicillin, 50 μg/ml streptomycin, and 10% (v/v) fetal bovine serum. For experiments, cells were used at passage four or less and maintained in medium with 10% serum. HeLa (LGC promochem, Teddington, UK) cells were cultured in minimum essential medium (Sigma, Dublin, Ireland) supplemented with 10% (v/v) fetal bovine serum, 2 mM L-glutamine, 50 U/ml penicillin, 50 μg/ml streptomycin, and non-essential amino acids (Sigma). Cos7 (LGC Promochem) cells were cultured in DMEM (Cambrex) supplemented with 10% (v/v) fetal bovine serum, 2 mM L-glutamine, 50 U/ml penicillin, and 50 μg/ml streptomycin.

For hypoxia experiments, cells were placed in an hypoxia chamber (Coy Laboratories, Grass Lake, MI) allowing the establishment of humidified, ambient, atmospheric hypoxia of 1% O_2_ with 5% CO_2_ and a balance of N_2_. Temperature was maintained at 37 °C. Extracellular pO_2_ measurements were made by using fluorescence quenching oxymetry (Oxylite-2000; Oxford Optronix, Oxford, UK).

### RNA extraction and cDNA synthesis

RNA was extracted from hRPC using RNeasy kit (Qiagen, Crawley, UK) according to the manufacturer’s instructions. RT–PCR was performed as follows: 2 μg of total RNA was treated with DNaseI (Invitrogen), according to the manufacturer’s instructions, to remove chromosomal DNA. Reverse transcription was performed using random primer (Invitrogen) and Superscript II (Invitrogen) using the manufacturer’s protocol.

Limited cycle PCR was performed using the following primers, designed using the Primer3 software described in (Table 1) [11].

**Table 1:** Limited cycle and real time PCR primers

**Limited cycle PCR primers**			
Gene	**Sense primer 5′-3′**	**Antisense primer 5′-3′**	**Probe 5′-3′**
*VEGF*	CTGCTCTACCTCCACCATGC	CTGCATTCACATTTGTTGTGC	
*CHL-1*	TCTGTCACCTTTTGCAGTGG	GTTGCCGATTCTGAA AGAGC	
*Cox2*	AACAGGAGCATCCTGAATGG	ATTTCATCTGCCTGCTCTGG	
*RTP801*	TCTTAGCAGTTCTCGCTGACC	TGGCACACAAGTGTTCATCC	
*BMP-2*	CAAGATGAACACAGCTGG	TTTGCTGTACTAGCGACACC	
*BMP-4*	GGCTGTCAAGAATCATGG	GCTCAGGATACTCAAGAC	
*18S*	GTGGAGCGATTTGTCTGGTT	CGCTGAGCCAGTCAGTGTAG	
**Real time PCR primers**			
**Gene**	**Sense primer 5′-3′**	**Antisense primer 5′-3′**	**Probe 5′-3′**
*VEGF*	GTGCCCACTGAGGAGTCCA	GTGCTGGCCTTGGTGAGGT	CATCACCATGCAGATTATGCGGATCAA
*Cox2*	AGCCCTTCCTCCTGTGCCT	CAGGAAGCTGCTTTTTACC	TGATTGCCCGACTCCCTTG
*CHL-1*	CGTAGCTGAAGGGCTCTTT	ACACGTTTCCCTCCGAACA	AAATCGGCAACCCAATCAAT

Limited cycle PCR was performed using primers, designed using Primer3 software [11]. For real-time, PCR probes were labeled with 5′-FAM and 3′-TAMRA as quencher with the exception of the ribosomal probe, which was labeled with 5′-VIC to facilitate multiplexing.

### Real-time (Taqman) polymerase chain reaction

Real-time (Taqman) polymerase chain reaction transcript levels were determined by real-time PCR using a PerkinElmer 7700 analyzer (Perkin Elmer Life Sciences, Shelton, CT). Probe and primer sequences were described in Table 1. Probes were labeled with 5′-FAM and 3′-TAMRA as quencher with the exception of the ribosomal probe, which was labeled with 5′-VIC to facilitate multiplexing. All results were normalized to 18S rRNA (pre-developed assay reagent, PerkinElmer Life Sciences).

### Gene microarray analysis

RNA isolation was performed as described in the previous section. In vitro transcription and microarray analysis were performed as follows: 5 μg of total RNA were annealed to 100 pmol of the T7-(dT)24 primer. Then first-strand cDNA synthesis was completed by incubating the mixture with 1 μl of Superscript II RT (200 units) at 42 °C for 1 h. Second-strand synthesis was performed in a total volume of 150 μl. Following purification, the cDNA was extracted with phenol chloroform, precipitated with ammonium acetate, and resuspended in RNase-free water. Synthesis of cRNA was performed by in vitro transcription. The amplified cRNA was purified with an affinity resin column. Biotin-labeled cRNA prepared from template cDNAs was fragmented and hybridized to the HG U133A array (Affymetrix, High Wycombe, UK). Arrays were then washed and fluorescently labeled before being scanned using a confocal scanner. Microarray Suite 5.1 software (Affymetrix) was used to analyze the relative abundance of each gene from the average difference of fluorescent intensities. Calculated signal/log ratios for each gene at all time points of hypoxia were compared to normoxia, and those genes with a value greater than 0.6 or less than –0.6 (1.5 fold difference) in all three biologic replicate experiments were examined further.

### Cloning of the chordin-like 1 promoter and open reading frame

A 1537 bp fragment of the *CHL-1* promoter was amplified using the Advantage2 PCR enzyme system (BD Biosciences, Oxford, UK) from a human genomic library EMBL3 SP6/T7 (HL 1067 J, BD Biosciences) with the following primers, sense 5′-GGA GAT AAC AAC CAG AGA GTA GTG G-3′ and, antisense 5′-GAA AAG GAG GTG AGG GAA GC-3′. The PCR product was immediately cloned into the TA cloning vector pCRII-TOPO (Invitrogen). The plasmid was restricted using the enzymes Kpn1 (NEB) and Xho1 (NEB), the resulting promoter fragment was subcloned into the reporter vector pGL3 Basic (Promega Southampton, UK).

The open reading frame of *CHL-1* was amplified using the Advantage2 PCR enzyme system (BD Biosciences) using human retinal pericyte cDNA as a template. A sense primer with a BamH1 site, 5′-GCT CGG ATC CGC CTG TCA CCT TTT GCA GTG GTC-3′, and an antisense primer with an EcoR1 site, 5′-TGC AGA ATT CGC TTA CAG TGG CCC TTT TCA GAT C-3′, were used to generate CHL-1 expression from a parent vector, pcDNA6/V5-His C (Invitrogen), designated pcDNA6 CHL-1/V5-His.

### Overexpression of hypoxia inducible factor-1α

Wild-type (WT) and double mutant (DM) constructs expressing HIF-1α were a gift from Thilo Hagen (University College London, London, UK). Prolines 564 and 402 were mutated to alanine, as these are the residues important for targeted degradation in normoxia [12]. Pericytes were transfected, according to manufacturer’s protocol, with 4 µg of each construct using 12 µl Fugene 6 (Roche, Dublin, Ireland). After 24 h following transfection, the medium was changed and the cells were incubated for a further 24 h. RNA was then isolated and analyzed by RT–PCR, or nuclear protein extracts were examined by western blot analysis to detect HIF-1α protein.

### Luciferase assays

HeLa cells were transfected with plasmids pGL3-CHL-1 or pGL3-HRE [13] along with DM-HIF-1α, using 6 µl Fugene 6 (Roche). Cells were transfected for 24 h, medium replaced with fresh and incubated for a further 24 h. Cells transfected with DM-HIF-1α were incubated in normoxia, and cells transfected without DM-HIF-1α were incubated in hypoxia. Luciferase gene reporter assay was performed according to the manufacturer’s instructions (Promega).

### Analysis of chordin-like 1 and bone morphogenetic protein −4 complex formation

Cos7 cells were transfected as described previously with either empty vector, pcDNA/V5-HisC, or with the CHL-1 expression vector, pcDNA6 CHL-1/V5-His. Cells were lysed, and whole cell extracts were made. Next, 50 µl of extract was incubated with 250 ng rhBMP-4 (R&D Systems, Abingdon, UK) for 1 h at 4 °C before incubation with 100 µl Ni-NTA magnetic beads, overnight at 4 °C with constant rocking. Ni-NTA magnetic beads had been previously washed with binding buffer, pH 8.0, that contained 50 mM NaH_2_PO_4_, 300 mM NaCl, 10 mM imidazole, and 0.05% Tween 20. Complexes were washed in wash buffer, pH 8.0, that contained 50 mM NaH_2_PO_4_, 300 mM NaCl, 20 mM imidazole, and 0.05% Tween 20, and eluted off the magnetic beads with elution buffer, pH 8.0, that contained 50 mM NaH_2_PO_4_, 300 mM NaCl, 250 mM imidazole, and 0.05% Tween 20. Samples were electrophoresed on SDS–PAGE gels, transferred to PVDF membranes, and probed for either V5-tagged CHL-1 or BMP-4.

**Table 2:** Differential gene expression in human retinal pericytes exposed to hypoxia.

**Number**	**Accession number**	**Description**	**SLR**
**Upregulated genes**			
1	NM_000076.1	cyclin-dependent kinase inhibitor 1C (p57, Kip2, CDKN1C)	1.7
2	NM_000963.1	prostaglandin-endoperoxide synthase 2 (prostaglandin GH synthase and cyclooxygenase, PTGS2)	1.43
3	NM_013332.1	hypoxia-inducible protein 2 (HIG2)	1.43
4	BC005254.1	Similar to C-type (calcium dependent, carbohydrate-recognition domain) lectin, superfamily member 2 (activation-induced), clone MGC:12289	1.4
5	NM_016109	PPAR(gamma) angiopoietin related protein (PGAR)	1.36
6	AF064238.3	smoothelin large isoform L2 (SMTN)	1.26
7	AF022375.1	vascular endothelial growth factor	1.26
8	NM_014883.1	KIAA0914 gene product (KIAA0914)	1.16
9	NM_015675.1	growth arrest and DNA-damage-inducible, beta (GADD45B)	1.13
10	NM_002166.1	inhibitor of DNA binding 2, dominant negative helix-loop-helix protein (ID2)	1.06
11	NM_017606.1	hypothetical protein DKFZp434K1210 (DKFZp434K1210)	1.06
12	BC005127.1	adipose differentiation-related protein, clone MGC:10598	1.03
13	NM_020142.1	NADH:ubiquinone oxidoreductase MLRQ subunit homolog (LOC56901)	1.03
14	NM_003734.2	amine oxidase, copper containing 3 (vascular adhesion protein 1, AOC3)	1
15	NM_006931.1	solute carrier family 2 (facilitated glucose transporter), member 3 (SLC2A3)	0.96
16	S73751.1	acyl CoA:cholesterol acyltransferase	0.93
17	AL048423.1	EST: integrin, beta 5	0.9
18	NM_003897.1	immediate early response 3 (IER3)	0.9
19	NM_005195.1	CCAATenhancer binding protein (CEBP), delta (CEBPD)	0.9
20	AL049176	DNA sequence from clone 141H5 on chromosome Xq22.1–23. Contains parts of a novel Chordin LIKE protein with von Willebrand factor type C domains.	0.9
21	NM_020639.1	ankyrin repeat domain 3 (ANKRD3)	0.9
22	NM_002912.1	REV3 (yeast homolog)-like, catalytic subunit of DNA polymerase zeta (REV3L)	0.83
23	NM_000954.1	prostaglandin D2 synthase (21kD, brain; PTGDS)	0.83
24	BC000125.1	Similar to transforming growth factor, beta 1, clone MGC:3119	0.76
25	AI078167	EST: nuclear factor of kappa light polypeptide gene enhancer in B-cells inhibitor, alpha	0.76
26	NM_005384.1	nuclear factor, interleukin 3 regulated (NFIL3)	0.76
27	NM_019058.1	hypothetical protein (FLJ20500), mRNA. RTP801	0.73
28	NM_003670.1	basic helix-loop-helix domain containing, class B, 2 (BHLHB2)	0.66
**Downregulated genes**			
1	NM_000903.1	diaphorase (NADHNADPH, cytochrome b-5 reductase, DIA4)	-2.16
2	NM_003234.1	transferrin receptor (p90, CD71, TFRC)	-1.53
3	NM_020299.1	aldo-keto reductase family 1, member B10 (AKR1B10)	-1.53
4	NM_018092.1	neuropilin (NRP) and tolloid (TLL)-like 2 (NETO2)	-1.23
5	M21692.1	class I alcohol dehydrogenase (ADH2) beta-1 subunit	-1
6	D45421.1	mRNA for phosphodiesterase I alpha	-0.86
7	AK021882.1	cDNA FLJ11820 fis, clone HEMBA1006445, highly similar to putative tumor supressor NOEY2	-0.76

The transcriptional response of human retinal pericytes to hypoxia was examined by microarray analysis, the SLR (signal-log ratio) is the mean value of three independent experiments.

### Western blot analysis

Western blot analysis was performed by standard procedures [14]. HIF-1α protein levels were detected using a 1:500 dilution of either anti-HIF-1α (Biomol) or anti-HIF-1α antibody (AbCam, Canbridge, UK). V5-tagged CHL-1 was detected using a 1:5000 dilution of the anti-V5 antibody (Invitrogen). BMP-2 and BMP-4 was detected using 1:500 dilutions of the anti-BMP-2 and anti-BMP-4 antibodies (R&D Systems). CHL-1 was detected using a 1:1000 dilution of anti-CHL-1 antibody (R&D Systems).

### Angiogenesis assay

Human umbilical vascular endothelial cells and human diploid fibroblasts were obtained (day 1) as cocultures in 24 well plates (AngioKit, TCS CellWorks Ltd., Buckingham, UK). Medium, with treatments or vehicle, was replenished on days 1, 4, 7, and 9. Treatments were recombinant human BMP-2, BMP-4, and CHL-1 (R&D Systems). In addition, 2 ng/ml VEGF and 20 μM Suramin (TCS Cellworks) were used as positive and negative controls respectively. Tubule formation was examined at day 11. Cells were fixed with ice-cold 70% ethanol and tubules were quantitated and visualized following fixing and staining for CD31 platelet-endothelial cell adhesion molecule 1 (PECAM-1) using a combined ELISA and histology kit (TCS CellWorks Ltd., UK) according to the manufacturer's instructions. Angiogenesis was quantitated by using anti-CD31 antibody coupled to a soluble substrate, ρ-nitrophenol phosphate, which permits quantitation by an optical density measurement. Plates were subsequently washed and stained using an insoluble substrate, 5-Bromo-4-chloro-3-indolyl phosphate (BCIP)/Nitroblue tetrazolium (NBT), for tubule visualization.

### Statistical analysis

Each result was representative of at least three independent experiments. All values are represented as the mean ±standard error of measurement. The Student *t*-test was used to determine statistical differences. Significance of results was indicated when p<0.05.

## Results

### Upregulation of chordin-like 1 in human retinal pericytes in response to hypoxia

The transcriptomic response of human retinal pericytes to hypoxia was examined. The mean signal–log ratio of three independent experiments is shown (Table 2). Using oxygen quenching oxymetry, we determined extracellular oxygen tensions at the level of the monolayer. Cells cultured in normoxia had a mean pO_2_ measurement of 140±2.79 mmHg, cells cultured in hypoxia had a mean pO_2_ measurement of 2.12±0.94 mmHg. The ambient pO_2_ in the hypoxia chamber was 6.21±0.08 mmHg (data not shown).

**Figure 1:**
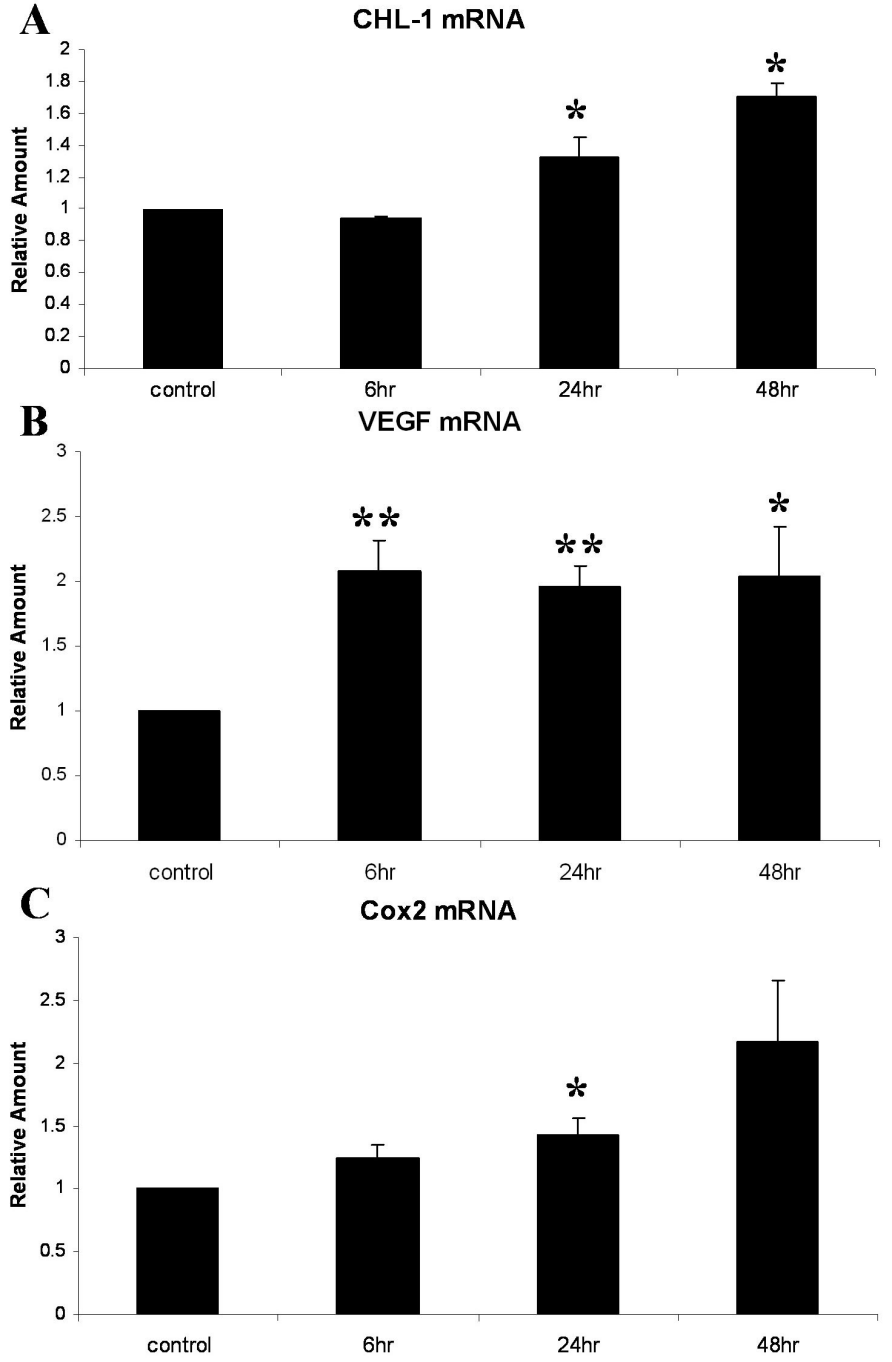
Validation of three genes differentially expressed in human retinal pericytes (hRPC) in response to hypoxia. The upregulation of a selection of genes was validated with real time PCR, using a PerkinElmer 7700 analyzer, on cDNA generated from human retinal pericytes exposed to increasing periods of hypoxia (0, 6, 24, and 48 h). All results were normalized to 18S rRNA, using a pre-developed assay reagent. Data are expressed as mean relative quantity of mRNA, relative to control, for three independent experiments ±standard error of measurement for (**A**) *CHL-1* mRNA, (**B**) *VEGF* mRNA, and (**C**) *Cox 2* mRNA. Data are expressed as mean±SEM values. The asterisk indicates a significance at p<0.05 and the double asterisk indicates a significance at p<0.001.

*CHL-1* (upregulated gene number 20) is a novel gene not previously associated with diabetic retinopathy [9,15,16]. *CHL-1* was upregulated in pericytes by hypoxia with a mean SLR of 0.9, corresponding to a 1.9 fold increase in expression (p<0.001). Two genes previously known to be associated with diabetic retinopathy were also upregulated: *VEGF* (upregulated gene number 7) is implicated in diabetic retinopathy, contributing to endothelial proliferation, permeability, and angiogenesis [2], and is known to be upregulated in pericytes in response to hypoxia [17]. *VEGF* was upregulated in pericytes by hypoxia with a mean SLR of 1.26, corresponding to a 2.4 fold increase in expression (p<0.001), and *Cox2* (upregulated gene number 2) is associated with neovascularisation in models of retinopathy [18,19]. *Cox2* was upregulated in pericytes by hypoxia with a mean SLR of 1.43, corresponding to a 2.7 fold increase in expression (p<0.05). To examine the upregulation of *CHL-1* along with genes known to be upregulated in diabetic retinopathy, we performed real-time quantitative PCR. As determined by quantitative PCR over a time course (0, 6, 24 and 48 h) of exposure to hypoxia (1% O_2_), *CHL-1* mRNA was upregulated 1.7 fold by 48 h (p<0.05; Figure 1A), and *VEGF* and *Cox 2* mRNAs were upregulated twofold by 48 h (p<0.05; Figure 1B,C).

### Chordin-like 1 expression is directly transactivated by hypoxia inducible factor-1α

The upstream region of *CHL-1* was identified using the Human Genome Browser Gateway at the UCSC interface. A 1537 bp fragment of the *CHL-1* promoter was cloned, sequenced and inserted into the reporter vector pGL3 Basic (Promega), and named pGL3 CHL-1. Bioinformatic analysis of transcription factors binding to this region of the promoter was performed using MatInspector^TM^ software, and a putative HIF-1α binding site was located in the proximal promoter (data not shown).

**Figure 2:**
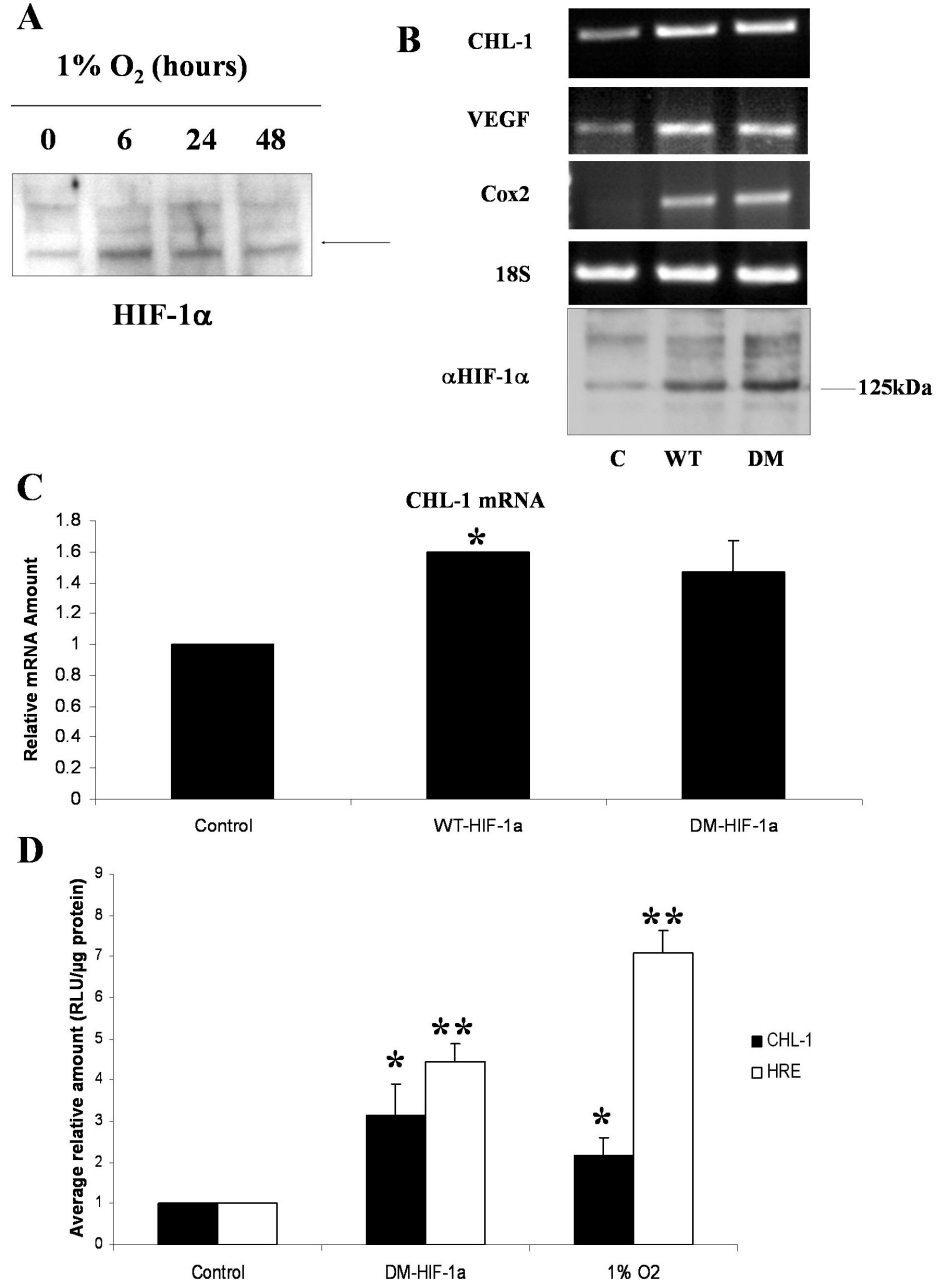
HIF-1α drives expression of chordin-like 1 in retinal pericytes exposed to hypoxia. **A**: western blot analysis of nuclear extracts generated from human retinal pericytes exposed to increasing periods of hypoxia (0, 6, 24, and 48 h) for HIF-1α shows upregulation of the protein by 6 h. **B**: Transfection of human retinal pericytes maintained in normoxia with expression vectors for HIF-1 α, C is control/empty vector, WT is wild type vector, WT-HIF-1 α, DM is double mutant vector, DM-HIF-1 α, using the transfection reagent Fugene6, induces expression of many of the genes upregulated in response to hypoxia, as measured by RT–PCR. 18S PCR is shown as a loading control and western blot analysis confirmed expression from each of the HIF-1α expressing plasmids. **C**: Induction of *CHL-1* mRNA in response to HIF-1α overexpression was quantitated by real time PCR. *CHL-1* levels were normalized to 18S rRNA, using a pre-developed assay reagent. Data are expressed as mean relative quantity of mRNA, to control, for three independent experiments ±standard error of measurement. **D**: HeLa cells were transfected, using the transfection reagent Fugene6, with the *CHL-1* promoter or a luciferase reporter construct containing four HIF-1α responsive elements (HRE), alone (control), cotransfected with the HIF-1α expression vector DM-HIF-1α, or alone and subsequent exposure to hypoxia for 24 h (1% O_2_). Cotransfection with DM-HIF-1α as well as exposure to hypoxia induced activation of the CHL-1 promoter and the HRE construct. Data are expressed as mean±SEM values. The asterisk indicates a significance at p<0.05 and the double asterisk indicates a significance at p<0.001.

Human retinal pericytes were exposed to 1% O_2_ for 0, 6, 24, and 48 h. Nuclear and cytosolic extracts were prepared and used for western blotting. HIF-1α protein expression was dramatically increased by 6 h of hypoxia and remained elevated by 48 h (Figure 2A).

To determine if the *CHL-1* promoter with the putative HIF-1α binding sequences was truly HIF-1α responsive, we transfected WT and DM constructs expressing HIF-1α into retinal pericytes. Semiquantitative RT–PCR was performed on RNA isolated from these cells for genes known to be HIF-1α responsive, *VEGF* and *Cox2*, and for the novel hypoxia regulated gene *CHL-1*. In addition, 18S rRNA PCR was performed as a loading control (Figure 2B). Expression of the transiently transfected HIF-1α is also shown in Figure 2. The level of *CHL-1* mRNA expression in these transiently transfected cells was quantitated using real-time PCR (Figure 2C). Overexpression of HIF-1α was confirmed by western blotting (Figure 2B).

To further determine the response of the *CHL-1* promoter to HIF-1α, we transfected HeLa cells with pGL3 CHL-1 and either stimulated with 1% O_2_ or transfected with pGL3 CHL-1 and DM-HIF-1α. As a control, the experiment was also performed using a promoter reporter construct containing four hypoxia responsive elements driving the luciferase gene, HRE-Luc, instead of pGL3 CHL-1. The *CHL-1* promoter was driven greater than threefold by HIF-1α (p<0.05) and greater than twofold by hypoxia (p<0.05). The control promoter reporter construct, which contains four hypoxia responsive elements (HREs) was driven greater than fourfold by HIF-1α and greater than sixfold by hypoxia (both p<0.001; Figure 2D).

### Chordin-like 1 expressed in human retinal pericytes is secreted and binds to bone morphogenetic protein-4

**Figure 3:**
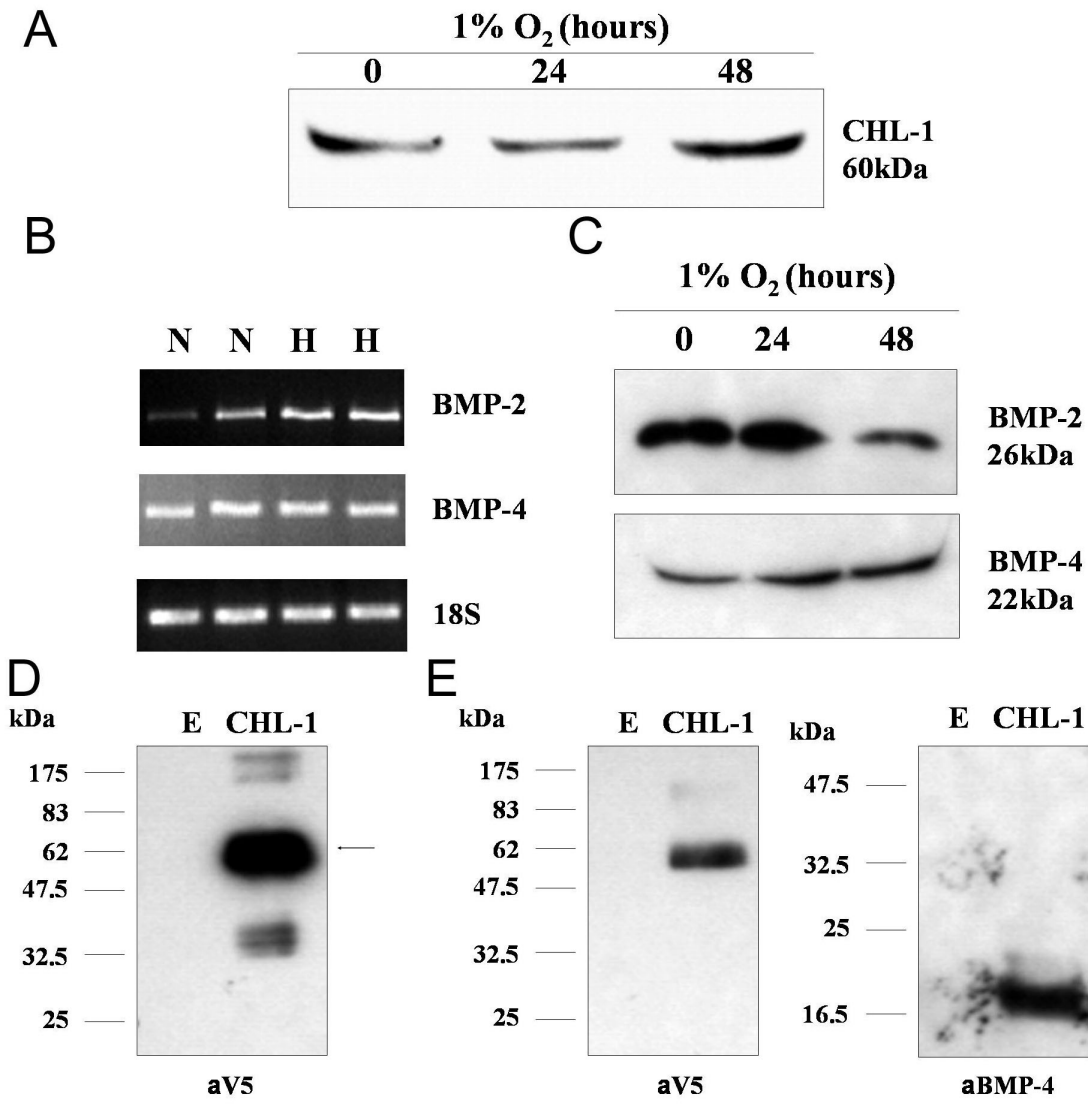
Chordin-like 1 expressed in human retinal pericytes is secreted and binds to bone morphogenetic protein-4. **A**: Conditioned media from HRPC exposed to 1% O_2_ for 24 and 48 h was examined by western blot analysis for secretion of CHL-1, using an anti-CHL-1 antibody. **B** and **C**: BMP-2 and BMP-4 expression in HRPC was examined in cells cultured in normoxia (N) and hypoxia (H) by RT–PCR (B) and secreted BMP2 and BMP-4 were detected in conditioned media from HRPC exposed to 1% O_2_ for 24 and 48 h (C). **D:** Transfection of the expression vector pcDNA6/CHL-1 V5-His into Cos7 cells, using the transfection reagent Fugene 6, resulted in expression of an approximately 60 kDa protein, which was detectable using an anti-V5 antibody. Cells were transfected with either an empty vector (E), pcDNA6/V5-His C, or a V5 tagged CHL-1 expressing vector (CHL-1), pcDNA6/CHL-1 V5-His. **E:** Whole cell extracts from Cos7 cells were transfected, using the transfection reagent Fugene 6, with empty pcDNA6/V5His (E), or with the expression vector pcDNA6 CHL-1/V5His (CHL-1) expressing V5His tagged CHL-1, were incubated with 250 ng rhBMP-4 and 100 ml NiNTA magnetic beads at 4 °C overnight. The complexes were washed, the beads and examined by western blotting for the presence of CHL-1, using anti-V5 antibody, and BMP-4, using an anti-BMP-4 antibody.

*CHL-1* mRNA was expressed in response to hypoxia in retinal pericytes (Figure 1C). The protein sequence for CHL-1 contained a signal peptide. Conditioned media (20 μl) from retinal pericytes exposed to hypoxia was examined by western blotting for secretion of CHL-1 using an anti-CHL-1 antibody (R&D Systems). Secreted CHL-1 was detectable in the conditioned media and was upregulated by hypoxia, correlating with the mRNA data (Figure 3A). CHL-1 is a BMP antagonist and previously shown to bind BMP-2 [20] and BMP-4 [9]. We investigated the expression of both these BMPs in retinal pericytes in response to normoxia and hypoxia. Both were expressed at the mRNA level (Figure 3B) and were secreted into the culture media as detected by western blotting (Figure 3C). Exposure to hypoxia had no significant expression on either BMP-2 or BMP-4 expression.

The open reading frame of *CHL-1* was cloned and sequenced, and inserted inframe with the COOH-terminal V5 6xHis tag in the expression vector pcDNA6/V5His, and named pcDNA6 CHL-1/V5His. Cos7 cells were transfected with empty pcDNA6V5His (E), or with the expression vector pcDNA6 CHL-1/V5His (CHL-1) expressing V5His tagged CHL-1. Whole cell extracts were examined by western blotting for V5 tagged CHL-1 expression using an anti-V5 antibody (Invitrogen; Figure 3D) Whole cell extracts from Cos7 cells transfected with empty pcDNA6/V5His (E), or with the expression vector pcDNA6 CHL-1/V5His (CHL-1) expressing V5His tagged CHL-1, were incubated with 250 ng rhBMP-4 (R&D Systems) and 100 ml NiNTA magnetic beads at 4 °C overnight with rotation. The interaction of the two proteins was demonstrated by pulling down any complexes formed with the tagged CHL-1 using NiNTA magnetic beads (Qiagen) which bound the 6xHis tag on the expressed CHL-1. Proteins bound in a complex to CHl-1 were washed with wash buffer and then eluted using elution buffer. Western blotting was used to identify eluted proteins. CHl-1 was detected using anti-V5 antibody (Invitrogen), and BMP-4, using an anti-BMP-4 antibody (R&D Systems). The complexes bound to the Ni-NTA magnetic beads contained both CHL-1 and BMP-4 (Figure 3E).

### Bone morphogenetic protein-4 inhibits angiogenesis and is antagonized by chordin-like 1

**Figure 4:**
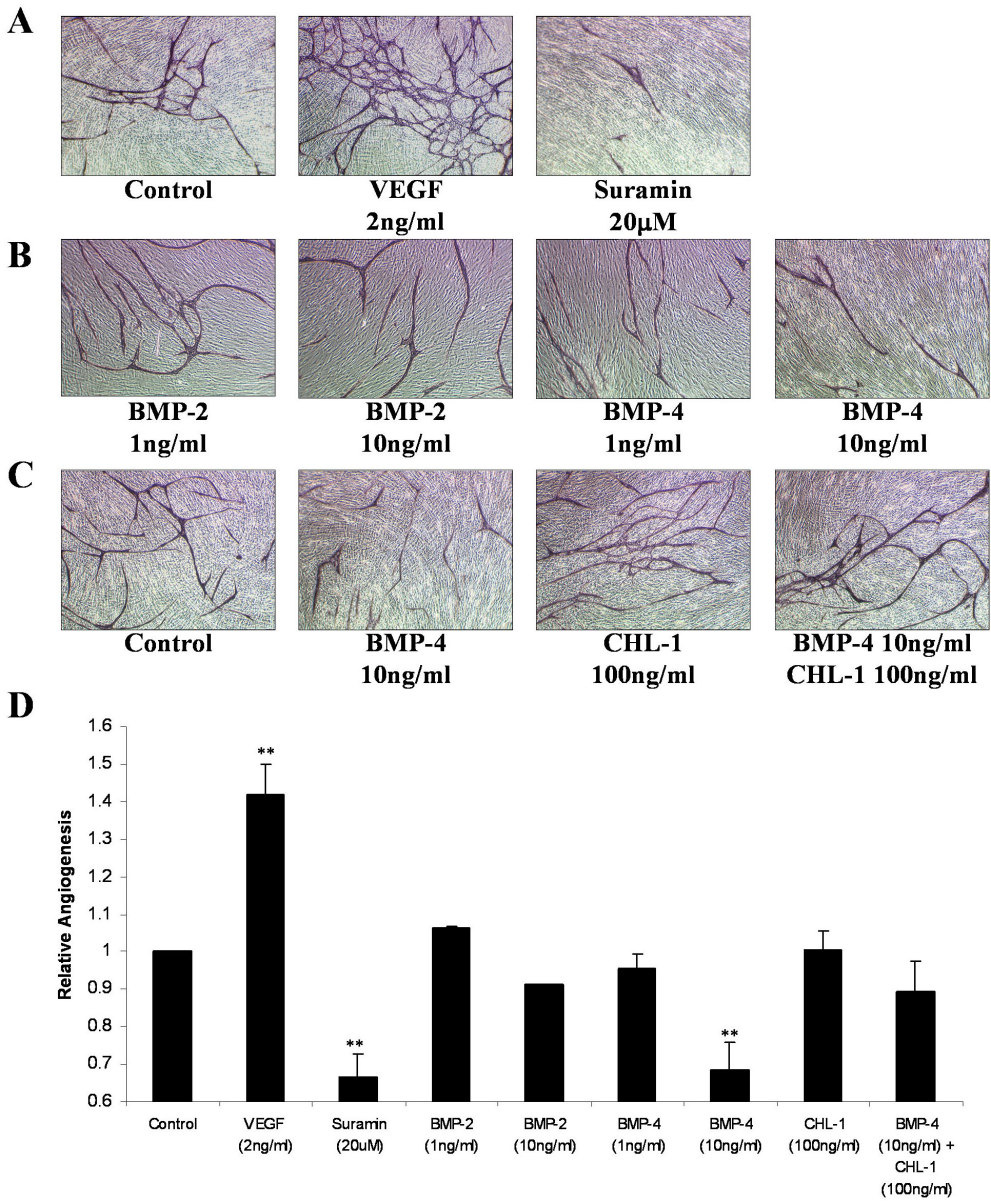
Chordin-like 1 modulates the antiangiogenic effect of bone morphogenetic protein-4. Human umbilical vascular endothelial cells(HUVECs) and human diploid fibroblasts were obtained (day 1) as cocultures in 24 well plates. Medium, with treatments or vehicle, was replenished on days 1, 4, 7, and 9. The assay was treated with VEGF, Suramin, recombinant human BMP-2, BMP-4, and CHL-1. Tubule formation was examined at day 11. Cells were fixed, quantitated, and visualized using a combined ELISA and histology kit. **A**: VEGF (2 ng/ml) and Suramin (20 mM) were used as positive and negative angiogenesis controls, respectively. B: Cells were treated with rhBMP-2 and rhBMP-4. BMP-4 significantly inhibited angiogenesis at 10 ng/ml. **C**: CHL-1 inhibited BMP-4; CHL-1 alone had no significant effect on angiogenesis, however it inhibited BMP-4s anti-angiogenic effects. Images **A-C** are shown at magnification 10X. Representative images are shown in **A-C**. **D**: Angiogenesis was quantitated by using anti-CD31 antibody coupled to a soluble substrate, ρ-nitrophenol phosphate (ρ-NPP), which permits quantitation by an optical density measurement. The asterisk indicates a significance at p<0.05 and the double asterisk indicates a significance at p<0.001.

**Figure 5:**
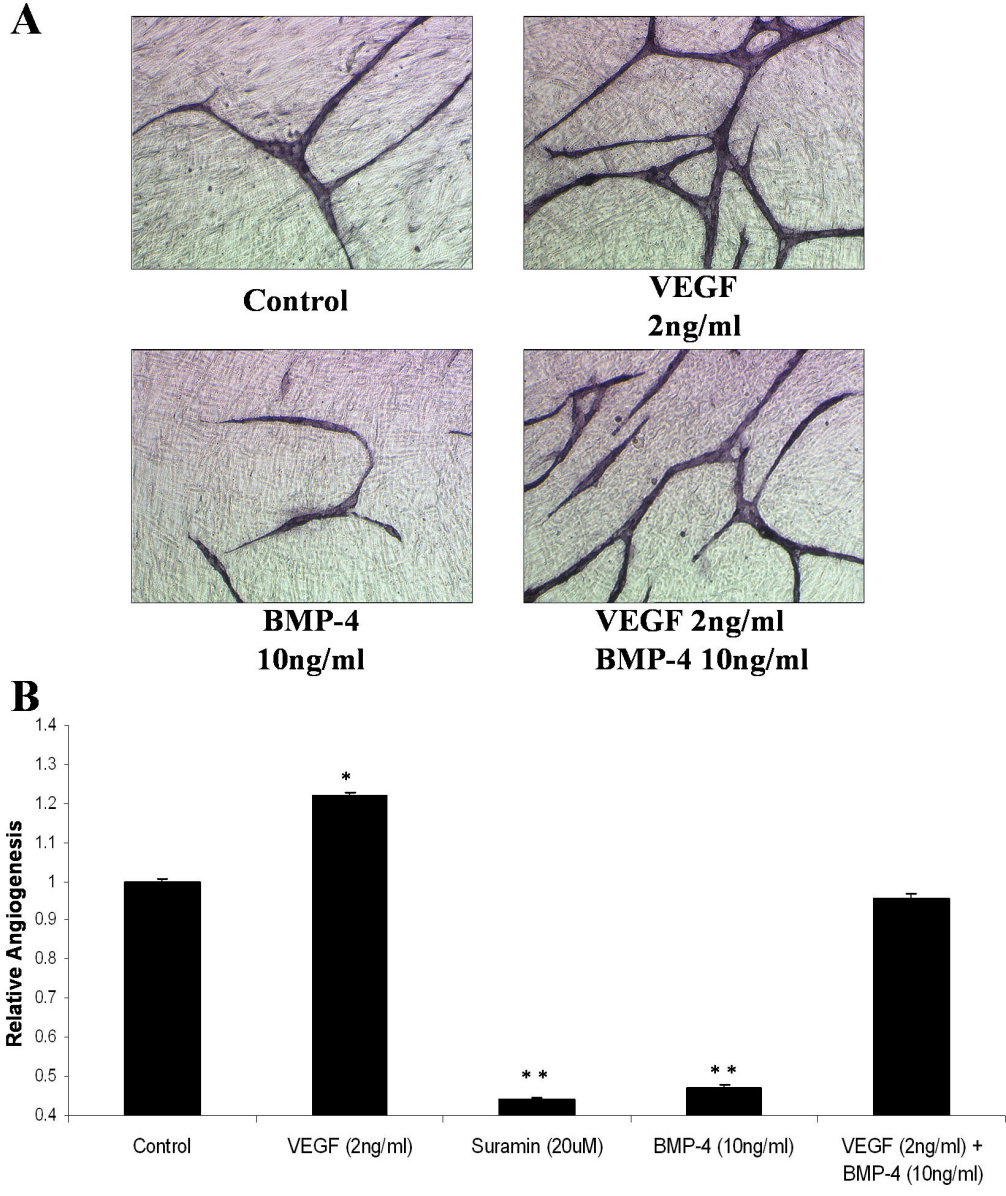
Vascular endothelial growth factor and bone morphogenetic protein-4 co-regulate angiogenesis. Human umbilical vascular endothelial cells (HUVECs) and human diploid fibroblasts were obtained (day 1) as cocultures in 24 well plates. Medium, with treatments or vehicle, was replenished on days 1, 4, 7, and 9. The assay was treated with vascular endothelial growth factor (VEGF), Suramin, and recombinant human BMP-4. Tubule formation was examined at day 11. Cells were fixed, quantitated, and visualized using a combined ELISA and histology kit. **A**: Angiogenesis assay demonstrating the combined effects of VEGF (pro-angiogenic) and BMP-4 (anti-angiogenic) on angiogenesis. Suramin was used as a negative control. (magnification 10X). Representative images are shown. **B**: Angiogenesis was quantitated by using anti-CD31 antibody coupled to a soluble substrate, ρ-nitrophenol phosphate (ρ-NPP), which permits quantitation by an optical density measurement. Data are expressed as mean±SEM values. The asterisk indicates a significance at p<0.01 and the double asterisk indicates a significance at p<0.001.

BMPs have been previously reported to be negative growth regulators in the adult retinal pigmented epithelium [21], and, more specifically, BMP-4 has been demonstrated to mediate apoptosis of capillary endothelial cells [22]. We wanted to determine the angiogenic effects of BMPs and CHL-1 using an in vitro angiogenesis assay (AngioKit, TCS Cellworks). Using 2 ng/ml VEGF and 20 μM Suramin as positive and negative controls, respectively, we successfully stimulated (p<0.001) and inhibited (p<0.001) angiogenesis (Figure 4A,D). While 10 ng/ml BMP-2 appeared to negatively regulate angiogenesis, this effect was not significant; however, 10 ng/ml BMP-4 significantly inhibited angiogenesis (p=0.0015; Figure 5B,D). CHL-1 alone had no effect on angiogenesis; however, it reversed the inhibitory effect of BMP-4 (Figure 4C,D).

### Vascular endothelial growth factor and bone morphogenetic protein-4 co-regulate angiogenesis

To examine whether the pro- and anti-angiogenic effects of VEGF and BMP-4 were mutually exclusive, we used these growth factors alone and in combination in an angiogenesis assay. We found 2 ng/ml VEGF stimulated angiogenesis and 10 ng/ml BMP-4 inhibited angiogenesis. In combination the two growth factors have no effect on angiogenesis (Figure 5).

## Discussion

The pathologic aberrations associated with diabetic retinopathy are localized primarily in the retinal capillaries. Pericyte loss and microaneurysm formation are hallmarks of early changes in the retinas of diabetic patients [23]. Retinal pericytes have been demonstrated to be involved in the regulation of endothelial cell number and morphology and microvessel architecture [8]. We have investigated the transcriptomic response of human retinal pericytes to hypoxic insult. Over three biologic replicate experiments, 35 genes (28 upregulated and seven downregulated) demonstrated differential expression after 48 h exposure to hypoxia. Among the upregulated genes were those whose expression has previously been associated with both hypoxia and diabetic retinopathy, such as *VEGF* [24–27] and *Cox2* [18,28]. One gene, *CHL-1*/ventroptin has been described in the context of the developing retina [9]; however, its regulation or an association with a disease process is yet to be described.

HIF-1α is a transcription factor implicated in hypoxia-elicited transcription. It binds HRE, a consensus element within promoters. We examined the promoters of the upregulated genes for the presence of the HIF-1α binding site (data not shown) and found 18 of the 27 upregulated genes contained HIF binding elements, including *CHL-1*. We also demonstrated increased nuclear presence of HIF-1α over a timecourse of exposure to hypoxia. Overexpression of HIF-1α drove the expression of many of the genes with HRE binding sites within their promoters, such as *VEGF*, *Cox2*, and *CHL-1*.

Our data demonstrate that CHL-1 (Ventroptin, Neuralin-1), a BMP antagonist, is expressed by retinal pericytes in response to hypoxic insult. Its promoter contains binding sites for many transcription factors, including the known hypoxia responsive transcription factors HIF-1α and cyclic AMP response element-binding protein (CREB; data not shown). We have demonstrated that *CHL-1* mRNA expression and the *CHL-1* promoter is driven by hypoxia and by overexpressing HIF-1α. This suggests that HIF-1α contributes to the expression of CHL-1 in a hypoxic environment. Another possible regulator of CHL-1 expression may be VEGF, as *VEGF* mRNA is upregulated by hypoxia by 6h, it is possible it contributes to the upregulation of CHL-1 expression by 24h, as activation of the VEGF receptors activates multiple signaling pathways [29]and the precise regulation of the *CHL-1* promoter has yet to be defined.

CHL-1 is a secreted protein expressed in the developing retina [9]. CHL-1 has three cysteine rich repeats (CRs). CRs are characteristic motifs that are conserved in some proteins including von Willebrand factor and chordin as well as other extracellular proteins, of which several are involved in regulation of BMP signaling regulation [30]. Because the three CRs of CHL-1 were significantly homologous to Chordin, Sakuta et al. [9] hypothesized and demonstrated that ventroptin (CHL-1) binds to and inhibits BMP-4. Another study demonstrated that ventroptin also inhibits BMP-2 [20]. This adds CHL-1 to a growing family of secretory proteins that antagonize BMPs, including chordin, Noggin, Cerberus, DAN, and Gremlin [31–34]. We have previously shown the BMP antagonist Gremlin to be upregulated in diabetic nephropathy [35,36] and localized to the outer retina of STZ diabetic mice [37]. Both BMP-2 and BMP-4 have been previously demonstrated to act as negative growth regulators in the retinal pigmented epithelium (RPE) [21]. We have found that human retinal pericytes express both BMP-2 and BMP-4. Other reports have shown decreases in BMP-4 in the retina in response to hypoxia [21]; however, in our study BMP-4 mRNA levels in retinal pericytes were unaffected by hypoxia. BMP-4 itself has been shown to promote apoptosis in the developing retina [38] and play a role in capillary apoptosis [22]. BMPs have also been demonstrated to stimulate angiogenesis through the production of VEGF [10,39]. We have shown that retinal pericytes not only express and secrete both BMP-2 and BMP-4, but they also secrete CHL-1 in response to hypoxic stimulus. Hypoxia is a well documented stimulus for angiogenesis and retinal neovascularization [40,41]. We have demonstrated here that hypoxia-induced HIF-1α drives CHL-1 expression in retinal pericytes, and that expressed recombinant V5-tagged CHL-1 binds BMP-4. Binding of CHL-1 to members of the BMP family has been previously demonstrated to modulate the function of the BMP [9]. Two studies showed regulation of angiogenesis by BMPs via *VEGF* [10,39]. More recently Vogt et al. [42] demonstrated that stimulation of ARPE-19 cells with BMP-4 results in increased VEGF expression. It has been documented that VEGFs secreted by epithelia, including the RPE, are likely to mediate paracrine vascular signals for adjacent endothelial (discussed in [43]). BMP-4 expression in the retina may have different effects on different cell types, such as increased VEGF expression from RPE cells and an anti-angiogenic effect on endothelial cells. In this study we demonstrated direct modulation of angiogenesis by BMP-4 in an in vitro angiogenesis assay and that this inhibition is modulated by the addition of CHL-1. We have also demonstrated that CHL-1 binds to BMP-4 and suggest that this binding prevents BMP-4 from acting at its receptor. Therefore, BMP-4 may play a role in the retina in maintaining capillary endothelial cell number, and the modulation of endogenous BMP levels in the retina may play a role in the plasticity of the retinal microvasculature in disease states. Induction of a BMP antagonist, namely CHL-1, by ischemic insult in a pathologic setting may function to neutralize BMP action on endothelial cells. Specifically, modulation of local BMP-4 activity in maintenance of capillary cell number by CHL-1, in response to a hypoxic environment, may contribute to retinal neovascularization, by allowing proliferation of endothelial cells. Gariano and Gardner [44] recently reviewed retinal angiogenesis and affirmed that pathological retinal neovascularization in the diabetic retina results from an imbalance of pro-angiogenic and anti-angiogenic factors. We have demonstrated in this study that angiogenic homeostasis may be maintained by such a combination, namely VEGF and BMP-4. We suggest that the balance between BMP-4 and VEGF is crucial in maintaining angiogenic homeostasis, and that the inhibition of the anti-angiogenic factor, BMP-4, by CHL-1, along with increases in VEGF tilt the balance in favor of a pro-angiogenic retinal environment.
